# Perception of Nigerian Dùndún Talking Drum Performances as Speech-Like vs. Music-Like: The Role of Familiarity and Acoustic Cues

**DOI:** 10.3389/fpsyg.2021.652673

**Published:** 2021-05-20

**Authors:** Cecilia Durojaye, Lauren Fink, Tina Roeske, Melanie Wald-Fuhrmann, Pauline Larrouy-Maestri

**Affiliations:** ^1^Department of Music, Max Planck Institute for Empirical Aesthetics, Frankfurt am Main, Germany; ^2^Department of Psychology, Arizona State University, Tempe, AZ, United States; ^3^Max Planck-NYU, Center for Language, Music, and Emotion, Frankfurt am Main, Germany; ^4^Neuroscience Department, Max Planck Institute for Empirical Aesthetics, Frankfurt am Main, Germany

**Keywords:** speech surrogacy, Yorùbá, categorization, amplitude modulation spectrum, pitch, intensity, timbre, timing

## Abstract

It seems trivial to identify sound sequences as music or speech, particularly when the sequences come from different sound sources, such as an orchestra and a human voice. Can we also easily distinguish these categories when the sequence comes from the same sound source? On the basis of which acoustic features? We investigated these questions by examining listeners’ classification of sound sequences performed by an instrument intertwining both speech and music: the dùndún talking drum. The dùndún is commonly used in south-west Nigeria as a musical instrument but is also perfectly fit for linguistic usage in what has been described as speech surrogates in Africa. One hundred seven participants from diverse geographical locations (15 different mother tongues represented) took part in an online experiment. Fifty-one participants reported being familiar with the dùndún talking drum, 55% of those being speakers of Yorùbá. During the experiment, participants listened to 30 dùndún samples of about 7s long, performed either as music or Yorùbá speech surrogate (*n* = 15 each) by a professional musician, and were asked to classify each sample as music or speech-like. The classification task revealed the ability of the listeners to identify the samples as intended by the performer, particularly when they were familiar with the dùndún, though even unfamiliar participants performed above chance. A logistic regression predicting participants’ classification of the samples from several acoustic features confirmed the perceptual relevance of intensity, pitch, timbre, and timing measures and their interaction with listener familiarity. In all, this study provides empirical evidence supporting the discriminating role of acoustic features and the modulatory role of familiarity in teasing apart speech and music.

## Introduction

When we turn on the radio it seems trivial to determine whether what we are hearing is music or someone speaking. Sound sequences can generally be described in terms of pitch, timbre, and timing (e.g., Kraus et al., 2009); acoustical differences have been shown between sequences associated with music and language domains. For instance, speech typically comprises many gliding tones and more variation in pitch trajectory than (Western) music (Patel, 2008), with a temporal modulation spectrum peaking around 5 Hz, approximating the syllable rate (Ding et al., 2017). On the other hand, music is characterized by discrete pitches sustained for longer durations (Zatorre and Baum, 2012), and a temporal modulation spectrum peaking around 2 Hz, approximating the average beat rate (Ding et al., 2017). However, note that such studies often examine material that has different sound sources, such as the human voice vs. musical instruments, which might enhance the distinctive characteristics of the sequences associated with each of the two categories.

When coming from the same source (e.g., the vocal instrument), music and language categories can show a certain overlap. For instance, certain types of speech are considered more musical than others (e.g., child-directed speech, rhymes, poetry), while certain types of vocal music are considered speech-like (e.g., rap). To better understand the ambiguity of vocal stimuli, Merrill and Larrouy-Maestri (2017) presented several versions of Arnold Schoenberg’s Pierrot lunaire—a piece notable for its use of sprechstimme or “speech-song” (Stadlen, 1981)—to vocal experts and found a large variety in the description of the material, from very spoken-like to very sung-like. Interestingly, the same exact material can be interpreted as either song or speech. A phenomenon called the speech-to-song illusion has been reported by Deutsch et al. (2008, 2011): A spoken sentence can be perceived as sung after several repetitions. In other words, the same acoustic contents can be perceived as belonging to two distinct categories, which makes it difficult to identify clear boundaries between speech and music, even in a culture in which the idea of contrasting categories is widely accepted (see Brown, 2000 for a gradual view of music-language). Further, it has been shown that categorization, in the context of the speech-to-song illusion task, changes when the material is difficult to pronounce (Margulis et al., 2015) or to understand (Jaisin et al., 2016), which supports the role of listeners’ familiarity or prior knowledge in stimulus categorization (Vanden Bosch der Nederlanden et al., 2015) and suggests a potential downside of using vocal stimuli, which are highly familiar to listeners.

In the present study, we focus on the perceptual categorization of non-vocal material into speech-like vs. music-like, as well as the role of familiarity in shaping these categories. Familiarity/culture effects on the perception of speech and music are well-described (Palmer and Krumhansl, 1987; Morrison and Demorest, 2009; Perrachione et al., 2011; Bregman and Creel, 2014; Sharma et al., 2020). With respect to timbre, familiar sound sources are recognized more quickly than unfamiliar ones (Siedenburg and McAdams, 2016). In the time domain, both rhythm (Hannon et al., 2012) and meter perception (Kalender et al., 2013) are altered as a function of listener familiarity with a stimulus type. Regarding pitch, Weidema et al. (2016) showed that the same contours are perceived differently depending on the context in which they are embedded, with better discrimination in music than speech context. These studies highlight that the role of specific acoustic features in shaping perceptual categorization may, in part, be driven by previous exposure.

Acoustically, sound sequences from music and language domains can be defined in several ways, with summary statistics (i.e., mean), as reported earlier, but also in terms of changes over time. In the case of pitch, for example, speech and music are rarely monotonous. In speech, the intonation or pitch direction (e.g., utterances interpreted as statements or questions, Bolinger, 1986; Ladd, 2008), the pitch accent (e.g., Ladd et al., 1999), or the prosodic patterns (e.g., Bänziger and Scherer, 2005; Kraljic and Brennan, 2005; Dilley and McAuley, 2008), play a considerable role in carrying paralinguistic information, such as the emotional state (Banse and Scherer, 1996) or the intention (Hellbernd and Sammler, 2016) of the speaker. Additionally, pitch contrasts and thus changes of pitch over time additionally carry lexical information in tonal languages (Yip, 2002; Carter-Ényì and Carter-Ényì, 2016). In music, changes of pitch over time also provide crucial information that allow listeners to recognize, evaluate, and enjoy a musical performance. There exist different musical (cultural) systems that define pitch movements, with specific scales and rules (Krumhansl, 1979; Lerdahl and Jackendoff, 1983; Cross, 2001; Ringer, 2002; Thompson, 2013), but some features such as the presence of small intervals or descending melodies are present in different cultures (e.g., Huron, 2001) and have been described as statistical universals for music (Savage et al., 2015). Besides the relevance of pitch changes over time (in addition to mean pitch), a large range of literature in the music and language domains supports the perceptual relevance of changes in other dimensions such as timbre, intensity, or duration. It is thus important to explore acoustic features that take into account dynamics (rather than means) not only with regard to pitch, but also more broadly in the dimensions of timing, timbre, and intensity.

In this study, we further examine speech-music specificities by employing an instrument intertwining language and music: the dùndún talking drum. The dùndún is commonly used in south-west Nigeria as a musical instrument. The dùndún of the Yorùbá is played by people of all ages, though mostly men, and in a variety of sacred and secular cultural contexts (Durojaye, 2020). It is used to play musical rhythms without semantic information but also to communicate announcements, warnings, prayer, jokes, proverbs, or poetry (Sotunsa, 2009). While a dùndún ensemble consists of drums of varying sizes and functions, we focus here on the ìyá ìlù dùndún—the lead drum in the ensemble—usually performing the role of “talker” during performance, imitating Yorùbá, which is a tonal language, in what has been described as speech surrogacy (Durojaye et al., in review; McPherson, 2018). Villepastour, in her analysis of bàtá drums—a very close relative of the dùndún—argues for the interdependence of speech tone and music and highlights the significance of relative pitch and rhythm in the surrogacy system of the dùndún (Villepastour, 2010, 2014).

**FIGURE 1:**
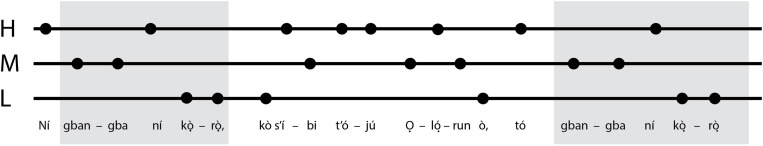
Transcription of a Yorùbá proverb played on the dùndún (stimulus 6S in our corpus). The dots placed on the three horizontal lines represent a schematic depiction of the pitch changes (high, medium, or low) as the phrase written underneath would be spoken in the Yorùbá language. The phrase translates to “Public or private, there is no place the God cannot see, public or private.” Though the phrase would typically be spoken without the repetition at the end, when played on the dùndún the proverb is elaborated by repetition of the first phrase (gray shaded areas), as repetition is often a means to provide context and remove ambiguity in meaning (Stern, 1957; Arewa and Adekola, 1980).

The Yorùbá language uses three relative tone levels: Low (grave accent), Middle (usually left unmarked), and High (acute accent). The tone levels are vital to distinguish the meaning of words (Carter-Ényì and Carter-Ényì, 2016). Like the language, the drum also consistently employs three relative tone levels. The dùndún is a waisted (hourglass shaped), double-headed membrane drum, with gut or leather cords securing the skins around the wooden frame of the drum. The cords are manipulated with one hand, while the other strikes the top membrane with a curved stick. Pressure on the cords changes the pitch of the drum, allowing for a full octave range and effects like glissandi (Blades, 1992; Euba, 1990). For the drum to produce the lowest pitch, minimal pressure is applied on the cords; the more the pressure, the higher the frequency. Thus, the drum can manipulate tone levels and contours, as in Yorùbá language. This imitation is confirmed by recent acoustical analyses of mono or disyllabic words performed on the drum which demonstrated that the three Yorùbá tones (Low, Middle, and High) are produced on a global level with three measurably different fundamental frequencies (Akinbo, 2019). The technique of representing syllables can take many forms such as (1) using one drum stroke for each syllable (as for a single tone level and vowel elisions); (2) many strokes for one syllable; (3) one drum stroke for two or more syllables; (4) one drum stroke for a syllable with many speech tone levels as would be the case for some glides, or assimilations (see also, Euba, 1990; Villepastour, 2010, for bàtá drums). A transcription of dùndún “talking” is provided as an example in Figure 1.

In the experiment outlined below, we seek to identify acoustic features associated with the perception of speech-like vs. music-like dùndún performances and the potential role of familiarity in such classification. To do so, we first compared speech-like vs. music-like dùndún performances with regard to different acoustic features related to pitch, intensity, timbre, and timing. Second, we presented the same samples to familiar and unfamiliar listeners and examined their ability to classify the performances as intended by the performer, as well as their confidence in the classification. Finally, a statistical model was created to quantify the role of listener familiarity and acoustic features of dùndún performance, in predicting listeners’ perception of dùndún as speech-like vs. music-like.

## Materials and Methods

The experimental procedure was in accordance with guidelines ethically approved by the Ethics Council of the Max Planck Society. Participants provided informed consent before proceeding with the study.

### Participants

One hundred and seven participants (36 self-reported as females, 71 as males, aged from 18 to 75 years old, *M* = 39.22, *SD* = 15.06) were recruited via the research participant database of the Max Planck Institute for Empirical Aesthetics and via personal contacts. From various geographical locations (15 different mother tongues were represented), fifty-one participants reported being familiar with the dùndún talking drum (i.e., they knew about the dùndún prior to the survey). Of these 51 participants, 28 (55%) were speakers of Yorùbá. Participants declared to have normal hearing ability and reported various musical training levels. Participants received no financial compensation.

### Material

Thirty-six dùndún samples were created from performances by one professional dùndún musician from Ibadan, South-West Nigeria. The performer (male) is a native Yorùbá and fluent English speaker with more than 25 years of experience playing the drum. Performances were recorded at a local music studio with a SHURE SM57 dynamic microphone directed at the face of the drum, at a 3-inch distance, sampling at 44.1 kHz. Note that clicks and environmental noise can be heard in some recordings.

Half of the performances were music-like material consisting of Yorùbá àlùjó rhythms (literally dance drumming); the other half were speech-like material, composed of Yorùbá proverbs and oríkì (poetry). The performer was first asked to use the drum to say different phrases (“talk”), after which he was asked to “drum” (the equivalent of music). All instructions were given in the Yorùbá language. Note that in Yorùbá dùndún performances, when drummers say they “talk” with the drum, they refer to the performance of oríkì, proverbs, or the signal mode of the drum. Similarly, when they talk about “drumming” or “playing music” (for those who use the English term), they are making a reference to àlùjó. These categories were confirmed by the performer after the task. Also note that dance rhythms, proverbs, and poetry are used for any occasion (e.g., weddings, burials, religious events). In the current performances, the “talking” contents covered various themes, such as a praise to a deity, prayers, vilification, and admonition.

To confirm that the performances clearly represented the category of speech or music, all recordings were presented to three independent professional dùndún drummers located in Nigeria and South Africa. The judges were asked to categorize the performances according to whether they represented speech or music. Like the performer, they used the terms àlùjó, oríkì, owe (proverbs), in their responses. The 30 samples on which the judges unanimously agreed to represent speech (*n* = 15) or music (*n* = 15) were selected. The duration of the samples ranged from 5 to 10 s (*M* = 7.37 s, *SD* = 1.1 s). All recordings can be accessed at: https://edmond.mpdl.mpg.de/imeji/collection/ovmWl7 rLtIiGSv1v.

### Procedure

The task was implemented as an online experiment in Unipark Enterprise Feedback Suite (QuestBack GmbH, Cologne, Germany). Prior to the classification task, a brief presentation of the dùndún was given (origin, uses, description, picture) without sound examples. The aim was to provide a basic knowledge for those who reported being unfamiliar with the drum or the potential use of this instrument as speech surrogates. To determine participants’ familiarity with the dùndún, we asked if they knew about the dùndún prior to the survey. Participants were instructed to listen to each excerpt and to indicate whether it was best described as “speech-like” or “music-like.” For each excerpt, the forced choice identification was followed by a confidence rating on a 4 point-scale (1 = not confident, 4 = very confident). The order of stimuli and response pattern (speech-like button as the first or the second option) was randomized for each participant.

### Acoustic Analyses

The analysis of acoustic features was carried out in MATLAB 2018b (The MathWorks, Inc., Natick, Massachusetts, United States).

#### Segmentation

Segmentation of single notes was performed semi-automatically on each recording’s amplitude envelope, using an adaptive threshold. First, amplitude envelopes were slightly smoothed with a Hodrick-Prescott (HP) filter (coefficient = 50). Then, the adaptive segmentation threshold was created by applying a stronger HP filter to the amplitude envelope (coefficient = #10^7^). The difference between the slightly smoothed amplitude envelope and the adaptive filter provided robust segmentation in most recordings. We visually inspected the segmented waveforms and sonograms while listening to the audio to validate segmentation. In cases where the automated segmentation had failed, we manually outlined note onsets with custom-written MATLAB code.

#### Acoustic Measures of Interest

First, we computed the amplitude modulation spectra of the recordings, following the procedure of Ding et al. (2017), with MATLAB code kindly provided by Nai Ding. In brief, we extracted the sound envelope in narrow frequency bands (corresponding to frequency bands used by the human cochlea), then, following a re-scaling procedure, we calculated the root mean square of the Discrete Fourier Transform of each frequency band and binned over frequencies. High frequencies in the amplitude modulation spectrum correspond to fast modulations of intensity, low frequencies to slow modulations (for details, see Ding et al., 2017). We then calculated the peak frequency in the spectrum (i.e., the frequency exhibiting the greatest amplitude modulation), for each recording. To further analyze differences in timing, we also computed the inter-onset interval (IOI, in ms) between notes, the two-interval ratio: interval1/(interval1+interval2), and the pulse clarity. The first measure corresponds to the timing at the note level and the second to the change in timing between consecutive time intervals. A short interval preceding a long interval would result in a ratio < 0.5, a short interval *following* a longer one has a ratio > 0.5, and an isochronous rhythm of two similar intervals has a ratio of 0.5. Pulse clarity, a measure that estimates the temporal regularity of events in the signal (Lartillot et al., 2008), was calculated using all recommended default parameters of the *mirpulseclarity* function from the music information retrieval toolbox v.1.7.2 (i.e., using a frame length of 5 s, a hop factor of 10%, and the maximum value of the autocorrelation curve to define clarity). Pulse clarity ranges between 0 (no clear pulse) and 1 (perfectly regular pulse).

Besides these time-related measures, we selected various features typically used to describe auditory signals: pitch, intensity, and timbre measures. At the *note level* and *between consecutive notes*, for each stimulus, we calculated: pitch height, intensity, and Wiener entropy (timbre, the maximum value is a pure sine tone). Scaling was performed within recording, on the millisecond-wise acoustic features between the 0.5 and 99.5th percentiles (instead of between minimum and maximum) to exclude outliers. Amplitude envelope and Wiener entropy were extracted from the audio in 10 ms time windows and steps of 1 ms using the MATLAB package Sound Analysis for MATLAB (by Sigal Saar). The pitch function from MATLAB Audio Toolbox (The MathWorks Inc., 2020) was used for pitch extraction. For pitch, intensity, and timbre measures (as well as IOI and ratio), we also calculated the probability densities for each group of stimuli (music-like and speech-like). Additionally, we computed the average of each feature, across each stimulus, resulting in the following final measures: mean pitch, mean intensity, and mean timbre; as well as average of absolute differences between consecutive tones (leading to mean intensity change, mean timbre change, and mean pitch change measures). Note that scaled values (0–1) were used to compute the measures capturing changes between consecutive notes.

### Behavioral Analyses

#### Participants’ Classification of Stimuli

Participants’ responses on the task can be summarized using a 2 × 2 contingency table, or confusion matrix M:

$$\text{M = }\left[\begin{array}{cc} \text{TP} & \text{FN}\\ \text{FP} & \text{TN} \end{array}\right]$$

where we arbitrarily define music as positive and speech as negative, such that true positives (TP) represent music intended by the performer and classified by the listener, while true negatives (TN) represent speech intended by the performer and similarly classified by listener. TPs (music) and TNs (speech) represent correct classifications. False positives (FP) designate instances of intended speech perceived as music, while false negatives (FN) indicate cases of intended music perceived as speech. Perfect classification is thus defined as 0 FPs and FNs: $\left[\begin{array}{cc} {\text{n}}^{+} & 0\\ 0 & {\text{n}}^{-} \end{array}\right]$. Accuracy on the task is defined as the number of correct classifications, divided by the total number of observations:

$$\text{accuracy}=\frac{\text{TP}+\text{TN}}{\text{TP}+\text{TN}+\text{FP}+\text{FN}}$$

However, accuracy has been shown to be biased if the classes are unbalanced (e.g., if there would be more instances of perceived speech than music). Therefore, as widely used and recommended, we computed Matthews Correlation Coefficient (MCC):

$$\text{MCC}=\frac{\text{TP}\times \text{TN}-\text{FP}\times \text{FN}}{\sqrt{\left(\text{TP}+\text{FP}\right)\times \left(\text{TP}+\text{FN}\right)\times \left(\text{TN}+\text{FP}\right)\times \left(\text{TN}+\text{FN}\right)}}$$

MCC is robust to unbalanced datasets, and has been shown to be a more reliable measure than accuracy (Chicco and Jurman, 2020). It is a measure of classification performance across all classes that takes into account the size of each class. MCC ranges between -1 and 1, with 0 indicating chance performance, 1 perfect performance, and -1 perfect misclassification. As is common practice with MCC, in the case of 0 in the denominator, we set the denominator to 1.

#### Statistical Analyses

Statistical analyses were conducted in R Core Team (2013). To compare acoustic measures between speech- and music-like dùndún recordings, we performed independent, two-tailed *t*-tests. For measures relative to changes between consecutive notes, absolute (rather than signed) differences were used. At an alpha of 0.05, the threshold *p*-value after correcting for multiple comparisons (10 *t*-tests) was *p* = 0.005. Cohen’s d is used to report effect sizes and was calculated in R using the cohen.d function in the *effsize* package (Torchiano, 2020).

To predict participants’ perception of the stimuli as music- or speech-like, a generalized linear mixed effects logistic regression model was fit via maximum likelihood using the Laplace approximation method, with bound optimization by quadratic approximation, implemented using the glmer function from the *lme4* package (Bates et al., 2015). Acoustic variables were centered and scaled before being entered into the model. Multicollinearity was checked using variance inflation factors (VIFs). Some predictors had a VIF > 5 and, therefore, required removal from the model. Specifically, inter-onset-interval (VIF = 5.90) and amplitude difference between adjacent notes (VIF = 8.27) were removed. Correlations between means of all acoustic features are provided in Supplementary Figure 1. Note that inter-onset interval has a high correlation with intensity [*r*_(28)_ = 0.63, *p* < 0.001] and intensity difference [*r*_(28)_ = −0.82, *p* < 0.001], and that intensity and intensity difference have a high correlation with each other [*r*_(28)_ = −0.71, *p* < 0.001].

## Results

### Acoustic Properties of Dùndún Performances

**FIGURE 2:**
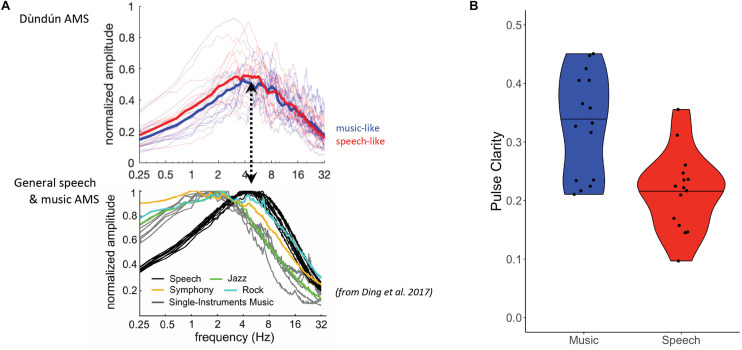
**(A)** Amplitude modulation spectra of the dùndún performances (top panel). Thin lines are for AMS of individual recordings, thick lines represent the mean for 15 music (blue) and 15 speech-like (red) stimuli. Bottom panel shows AMS for speech and music corpora from Ding et al. (2017) for comparison. Dashed arrow illustrates that dùndún recordings peak at higher modulation rates than most Western music, namely rates that are typical for speech (black line) but also occur in rock music (teal line). The lower panel in A is reprinted from Neuroscience and Biobehavioral Reviews, 81, Nai Ding, Aniruddh D. Patel, Lin Chen, Henry Butler, Cheng Luo, David Poeppel, Temporal modulations in speech and music, 7, Copyright Elsevier Ltd. (2017), with permission from Elsevier. **(B)** Violin plots representing the pulse clarity of the music (blue) and speech-like (red) stimuli. The black horizontal line indicates the median of both distributions. Black dots represent pulse clarity for individual stimuli.

We first investigated whether the amplitude modulation spectrum (AMS) systematically differs between speech-like and music-like dùndún stimuli, as the AMS has previously been shown to distinguish between speech (around 4–6 Hz) and different kinds of Western music (around 2 Hz). As illustrated in Figure 2A and confirmed via *t*-test, however, the AMS of the two types of stimuli did not significantly differ [*t*_(__27.93)_ = 1.60, *p* = 0.120, *d* = 0.59]; both peaked around 5 Hz, which corresponds to the previously established amplitude modulation rate typical of *speech* (Ding et al., 2017; Figure 2A, bottom panel). In terms of pulse clarity, we find a significant difference between the two stimulus categories, with a greater pulse clarity (i.e., greater temporal regularity) in the music-like stimuli, *t*_(__26.14)_ = 4.03, *p* < 0.001, *d* = 1.47 (Figure 2B).

**FIGURE 3:**
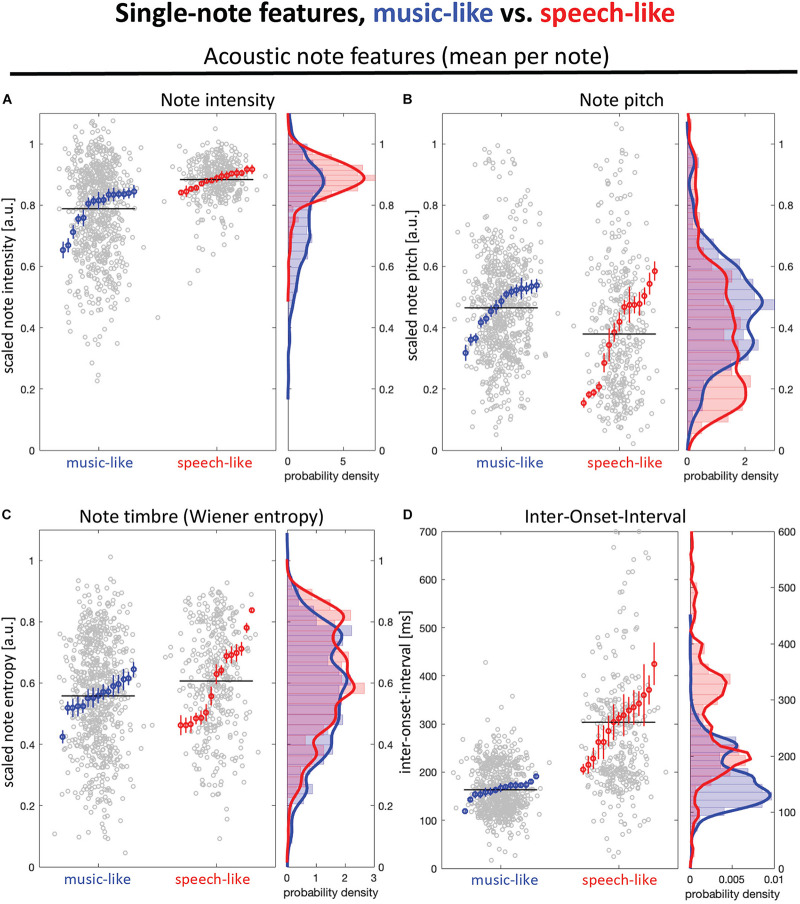
Acoustic measures computed at the note level for music-like (blue) and speech-like (red) dùndún performances. **(A)** Mean intensity, **(B)** Mean pitch, and **(C)** Mean timbre (Wiener entropy) **(D)** Mean inter onset intervals (IOI). Left panels show mean note values for 15 speech- and music-like pieces each in gray, with mean and SEM per piece in color. Right panels show the respective probability densities.

**FIGURE 4:**
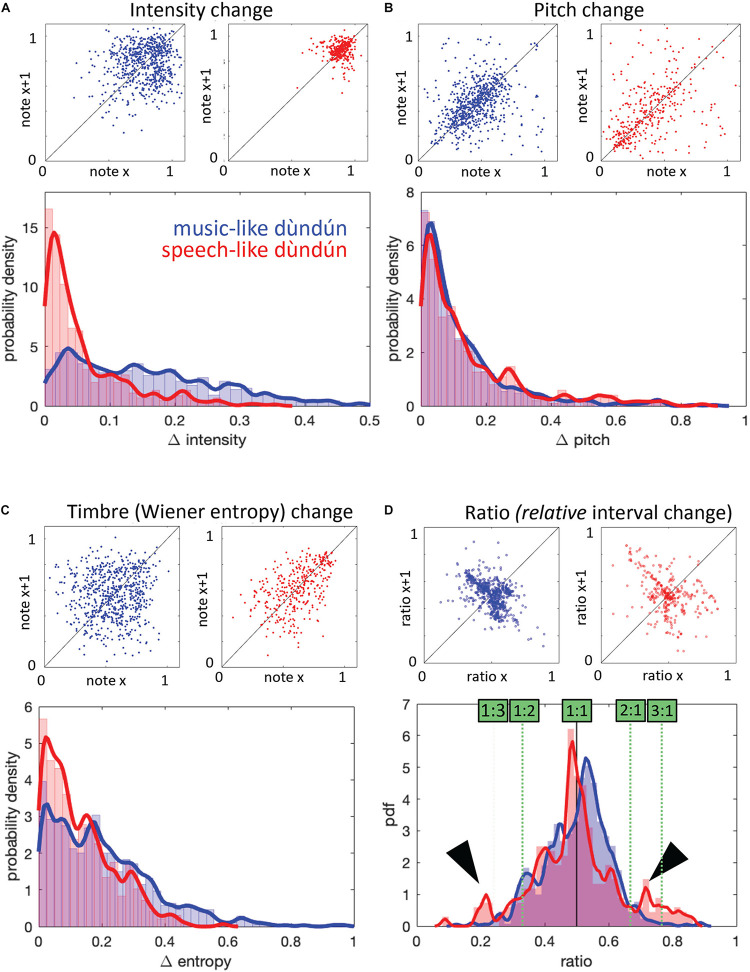
Acoustic measures computed at the level of consecutive notes for music-like (blue) and speech-like (red) dùndún performances. All measures are scaled from 0 to 1. **(A)** Mean intensity change: The top panel shows, for each pair of consecutive notes, the intensity of the pair’s first note on the *x*-axis, and of the second note on the *y*-axis. The diagonal line indicates where note pairs with two similar note intensities would fall. The bottom panel shows the probability density of intensity differences. **(B,C)** are the Mean pitch change and Mean timbre change, illustrated following the same logic as **(A)**. **(D)** Similar illustration for change in IOIs, as measured by ratio = interval1 / (interval1+interval2). In the probability density plot (bottom), green boxes and dashed lines mark interval ratios in terms of “interval1: interval2,” an alternative way to describe interval ratios, commonly used in music theory.

In addition to the AMS measure, we examined four types of features (see section “Materials and Methods” for description of all measures) that could capture differences between music- and speech-like stimuli: intensity, pitch, timbre, and timing. Figure 3 illustrates each measure at the note level (mean for each stimulus and their distribution). As reported in Table 1, we observed significant differences for intensity and timing, with higher intensity level (Figure 3A) and longer IOI (Figure 3D) in the speech-like recordings. Figure 4 illustrates each measure at the consecutive note level (mean of differences between consecutive notes for each stimulus and their distribution). Besides being louder, consecutive notes also varied less in intensity in the speech-like stimuli (Figure 4A), in line with the distribution depicted in Figure 3A, with a narrower range for the speech-like stimuli. Also, we observed that near-isochrony is very common in both speech- and music-like excerpts (Figure 4D). However, in music-like ones, intervals tend to speed up (the second interval in an isochronous pair being a little shorter, moving the near-isochronous peak slightly right) while slowing down in speech-like ones (near-isochronous peak moved slightly left). Some very high and very low ratios (due to a short interval next to a long one) become apparent in the wider spread of speech-like data, in the top right scatter plot, and in the peaks of the probability density plot, marked by arrowheads.

**TABLE 1:** Output of the independent *t*-tests carried out for the four types of acoustic features (Intensity, Pitch, Timbre, and Timing) between music- and speech-like stimuli.

	Intensity	Pitch	Timbre	Timing
Mean for note level	*t*_(__18.18)_ = −5.37	*t*_(__20.61)_ = 2.06	*t*_(__18.84)_ = −1.38	*t*_(__16.18)_ = −8.53
	*p* < 0.001	*p* = 0.052	*p* = 0.185	*p* < 0.001
	*d* = −1.96	*d* = 0.75	*d* = −0.50	*d* = −3.12
Mean change for consecutive notes	*t*_(__26.32)_ = 8.95	*t*_(__17.53)_ = −0.75	*t*_(__27.60)_ = 4.09	*t*_(__15.74)_ = 2.17
	*p* < 0.001	*p* = 0.465	*p* < 0.001	*p* = 0.046
	*d* = 3.27	*d* = −0.27)	*d* = 1.49	d = 0.79

*For the four types of comparisons, measures consisted of the mean of all notes (Figure 2) and the mean of the absolute difference between all consecutive notes (Figure 3). Note that the threshold p-value (for an alpha of 0.05) after correcting for multiple comparisons was p = 0.005. Thicker frames highlight the measures significantly differing after correction.*

### Listeners’ Classification of Dùndún Performances

**FIGURE 5:**
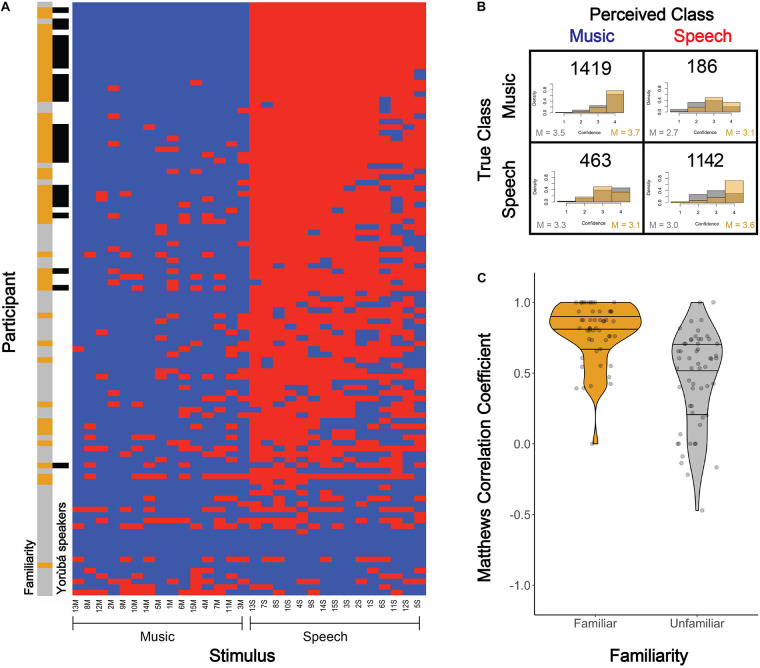
Participants’ classification of music vs. speech-like dùndún performances. **(A)** Each participant’s (vertical axis) judgment of each stimulus (horizontal axis) as music (blue) or speech (red). Participant performance is sorted descending from least to most errors. Columns are sorted from left to right, within each category (speech and music), from least to most errors per stimulus. The color bar to the left of the plot indicates whether each participant was familiar (golden) or unfamiliar (gray) with the dùndún and whether they spoke Yorùbá (black) or not (white). **(B)** Confusion matrix for perceived vs. intended stimulus classes, with histograms of participants’ confidence ratings (1–4) for each response type (i.e., quadrant), grouped by familiarity (gold = familiar; gray = unfamiliar). Confidence rating densities were computed within each response type (quadrant) for each familiarity group separately. Means for unfamiliar (gray) and familiar (gold) groups are displayed in the lower left and right corners of each quadrant, respectively. **(C)** Violin plots and underlying data points indicating the Matthews Correlation Coefficient (MCC) for each participant, separated according to those who were familiar with the dùndún (golden) vs. unfamiliar (gray). The bottom and top horizontal black lines in each distribution represent the 25th and 75th percentiles, the middle line represents the median. An MCC of 1 indicates perfect classification; 0 represents chance, and -1 perfect misclassification.

As can be seen in Figure 5A, participants clearly separated the stimuli into two distinct speech vs. music categories that largely aligned with the intention of the performer. We observed that only four participants categorized every sample as music (solid blue rows near the bottom of the plot), whereas the large majority showed few confusions. Twelve participants exhibited perfect classification (top rows). In the figure, within the speech and music categories, stimuli (columns) are sorted by the number of errors made per stimulus (i.e., the left-most column, stimulus 13M, was least often confused for speech, while 3M was most often confused for speech). Within the speech category, 13S was most clearly perceived as speech, while 5S was most often confused for music. Readers can access all stimuli online (link in section “Materials and Methods”).

A confusion matrix for perceived vs. intended music and speech-like performances is plotted in Figure 5B. Overall, the average accuracy of participants on the task was 66%. The average rate people perceived speech when the performance was intended to be music was 12%, while the average rate at which people perceived music when it was intended to be speech was 29%. Collectively, these latter two rates indicate that participants were more likely to perceive speech as music than music as speech. The illustration of confidence ratings (underlying histograms in Figure 5B, with ratings from 1 to 4) showed similar patterns, with moderately high confidence even in the case of false classifications. Note, however, that the listeners who were unfamiliar (gray) with the dùndún seem to be least confident when they perceive the stimulus to be speech (right column in confusion matrix). Confidence means for unfamiliar (gray) and familiar (gold) participants are displayed in the lower left and right corners of each quadrant, respectively.

Given the imbalance in perceiving speech vs. music, and the statistical properties outlined in the methods, our main metric of interest for participants’ classifications of the stimuli was the Matthews Correlation Coefficient (MCC). An MCC of 1 indicates perfect classification, 0 chance, and -1 perfect misclassification. Participants’ average MCC was 0.61 (±0.33). Participants who were familiar^1^ with the dùndún exhibited a significantly higher MCC, compared to those who were unfamiliar with the dùndún [Welch’s independent two-tailed, *t*-test: *t*_(__93.08)_ = 6.12, *p* < 0.001, Cohen’s *d* = 1.16, ΔMCC = 0.33]; see Figure 5C; N.B. each participants’ familiarity (gold = familiar; gray = unfamiliar) is plotted in the left color bar of Figure 5A). Nonetheless, familiarity is not required to perform the task, as unfamiliar participants still exhibited an average MCC of 0.46, well above chance (0), *t*_(__55)_ = 10.10, *p* < 0.001, *d* = 1.35.

### Predictors of Listeners’ Perception of Speech vs. Music

In an effort to understand which acoustic features were most relevant in participants’ perception of the dùndún excerpts as music vs. speech-like, we built a linear mixed effects logistic regression model. The binary dependent variable was participants’ perception (speech = 0, music = 1). On the stimulus level, fixed effects included all measures reported in Figures 2–4, except intensity difference and inter-onset-interval, which had to be excluded due to high correlation with intensity and each other (see section “Materials and Methods”). Since we observed an effect of familiarity on the classification performance index, with better classification for listeners who were familiar with the dùndún, we included familiarity as a fixed effect and in interaction with all acoustic measures. Confidence ratings were also entered as fixed effects. Random intercepts were included for participants and stimuli.

**FIGURE 6:**
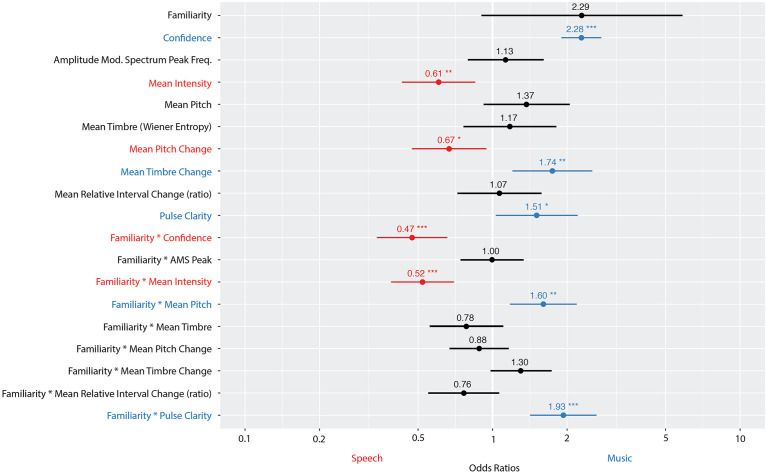
Odds ratios, with confidence interval (CI), for each fixed effect in a logistic regression model predicting participants’ perception of stimuli as music-like (1) or speech-like (0). The vertical line at 1 indicates no effect (i.e., any fixed effect predictor whose odds ratio CI overlaps 1 does not significantly predict participants’ perception). Fixed effects with an odds ratio <1 (red) indicate that a high value on that feature leads to the perception of speech. Odds ratios >1 (blue) indicate that a high value on that feature leads to the perception of music. The significance of each fixed effect is indicated with stars (***p < 0.001, **p < 0.01, and *p < 0.05). Number of observations: 3,210. Familiarity was a binary predictor (0 = no; 1 = yes); confidence ranged from 0 to 3; all other variables were continuous.

Figure 6 shows the odds ratios and confidence intervals for each fixed effect in the model. Fixed effects with an odds ratio <1 (red) indicate that a high value on that feature leads to the perception of speech. Odds ratios >1 (blue) indicate that a high value on that feature leads to the perception of music. Overall, the model had a prediction accuracy of 85% and an MCC of 0.70. The model explained a significant proportion of variance in the data, with a marginal R^2^ of 0.46 (amount of variance explained by fixed effects alone) and a conditional R^2^ of 0.65 (amount of variance explained by fixed and random effects).

Greater pulse clarity predicted perception of music. At the note level, greater mean intensity predicted speech. In terms of changes between notes, greater changes in pitch predicted speech, while greater changes in timbre predicted music. However, mean intensity, pitch and pulse clarity all interacted with participants’ familiarity (same direction of the overall effects but enhanced magnitude). Additionally, confidence predicted the categorization “music-like,” and interacted with familiarity, such that those familiar and confident were more likely to classify a given stimulus as speech, while those not familiar and confident were more likely to classify the stimulus as music. These confidence/familiarity results are in line with the general trends presented in Figure 5B.

## Discussion

In this study, we used an instrument capable of speech surrogacy to explore the boundaries between speech and music. While several instruments such as trumpets (Kaminski, 2008), flutes (Moore and Meyer, 2014), xylophones (Strand, 2009; Zemp and Soro, 2010; McPherson, 2018), and whistling (Stern, 1957) can produce speech surrogates, we focussed here on the dùndún talking drum to examine listeners’ perception of music and speech and, more specifically, the role of acoustic features in distinguishing these two categories. To do so, we recorded expert dùndún performances, which have the advantage of being able to create both musical and speech-like stimuli without requiring the human voice (highly familiar to all listeners). Acoustic measures relative to pitch, timbre, intensity, and timing were used to describe the stimuli and we measured listeners’ ability to classify each performance into two pre-defined categories intended by the performer: speech- or music-like.

Participants could accurately classify the dùndún performances in the category intended by the performer, with an unsurprising bias toward the music-like category, given that drums are more commonly associated with music than speech. Listeners who were familiar with the instrument seem to have a clearer representation of what is grouped into speech or music categories, as visible by their better classification performance. Such results could be driven by the fact that slightly over half of the familiar participants also reported speaking Yorùbá, though the familiarity effect remained even when Yorùbá-speaking participants were removed from the analysis. In any case, if familiarity (broadly defined) or Yorùbá fluency sharpens the categories, it is not required to perform the task, as shown by the above chance level MCC and relatively high confidence for participants who were not familiar with the instrument. Such results suggest the relevance of commonly recognized acoustic cues that shape each category but become fine-tuned through repeated exposure.

In the current study, we asked broadly about listeners’ familiarity and thus are not able to disentangle what exactly underlies the familiarity effect. Teasing apart perceptual vs. cultural vs. linguistic familiarity might all be of interest in future research. Additionally, manipulation of familiarity, via priming or additional explicit information about the dùndún, might allow one to quantify the amount and type of previous exposure that affects the classification. Further, exploration of listeners’ perception of dùndún performances with less constrained answer types might reveal other categories that could include both music and speech-like performances or less strong boundaries between these two culturally shaped categories (Brown, 2000).

The model proposed to examine the predictors of participants’ classification revealed that participants relied on several features. Pulse clarity, mean note intensity, and mean timbre change between consecutive notes were significant predictors. Additionally, familiarity seemed to amplify the effects of pulse clarity and intensity in predicting music vs. speech, respectively. These perceptual results are in line with those we find to distinguish acoustically between the two different stimulus categories. However, we also observed that features which did not significantly differ between the speech and music recordings nonetheless contributed significantly to the perceptual model. Specifically, mean pitch did not differ acoustically between the two types of stimuli performed by the musician, though it interacted with familiarity in predicting the perception of music. Similarly, mean pitch change between consecutive notes did not significantly differ acoustically but was associated with the perception of the stimuli as speech. In the future, the nature of the music and speech categories (that are slightly modulated by the familiarity of the listeners) could be clarified by means of psychophysical experiments that parametrically manipulate the relevant acoustic features reported here.

It is interesting to note the considerably faster amplitude modulations of the dùndún performances, compared to the AMS previously established for music (around 2 Hz, Ding et al., 2017) or to the perceived rate in natural sounds (Roeske et al., 2020). In the current study, peaks stand around 5 Hz for both music and speech-like dùndún. Though some musical styles have been shown to also peak around 5 Hz (e.g., rock), this rate is consistent with the modulation rate of speech (Ding et al., 2017). However, while Ding et al. (2017) analyzed a variety of Indo-European languages (American and British English, French, German, Swedish, Dutch, Danish, Norwegian; exception: Chinese), they did not include any Niger-Congo languages, such as Yorùbá, and only included Western types of music, which limits the generalizability of their findings. Here we extend Ding et al.’s AMS analysis to non-Western stimuli (dùndún) and show that its peak closely resembles that of speech. Future work might extend the AMS analyses to spoken Yorùbá and compare with that of the dùndún to better understand the findings about the temporal aspect reported here. Note also that, while mean IOI was significantly different between the two types of stimuli, it unfortunately could not be included into our statistical model (like the mean intensity change measure), as it had high correlation with other features (Supplementary Figure 1). Thus, it could well be the case that participants are using IOI (as well as intensity changes) in their classification. This issue could be addressed by systematically manipulating IOI, as well as mean intensity and intensity change, to tease apart their perceptual relevance.

Regarding the dynamic aspect of timing, the present study focused mainly on consecutive notes or intervals, though we did include a measure of pulse clarity. Our pulse clarity metric was related to maxima in the autocorrelation function of our stimuli (i.e., periodic repetitions), but our measure set could also be extended to perceived beat and/or meter (e.g., Tomic and Janata, 2008; London et al., 2017) or the detection of repeated patterns (e.g., via recurrence quantification analysis Fukino et al., 2016 or multi-fractal analysis Roeske et al., 2018). The analysis/evaluation of longer stimuli would allow for application of a more extensive set of timing measures to investigate their role in speech vs. music distinction. Also, though our stimulus set is novel in that it consists of speech-like and music-like performances on the dùndún from the same performer, it is also limited in scope. Future studies might consider developing larger corpora with more examples of speech-like and music-like material from multiple performers. In addition, future research might also more closely consider the relationship between measures like IOI, perceived beat and meter, and AMS. Though IOI, AMS, and perceived pulse / meter all have previously reported preferred temporal ranges, which broadly seem to align with each other (e.g., Fraisse, 1963; Parncutt, 1994; Farbood et al., 2013; Gotham, 2015; Ding et al., 2017), it is likely that IOI, AMS, and perceived pulse / meter do not form a trivial and/or consistent relationship to one another across all timescales.

Previous studies have suggested that surrogate languages or language-based music, such as talking drums, may enhance our understanding of music and language (Patel, 2008; Winter, 2014). In the present study, such ecologically valid material provided the unique opportunity to compare stimuli coming from the same sound source (and performer) while representing different conceptual domains, which paves the way to a more in-depth understanding of speech/music differences/similarities. Altogether, our findings confirm the relevance of acoustic features relative to intensity, pitch, timbre, and timing in distinguishing speech and music, as well as the role of culture and/or exposure in defining such categories.

## Data Availability Statement

The datasets presented in this study can be found in the links below: https://edmond.mpdl.mpg.de/imeji/collection/ovmWl7rLtIiGSv1v, https://github.com/lkfink/Dundun.

## Ethics Statement

The studies involving human participants were reviewed and approved by Ethics Council of the Max Planck Society. The participants provided their written informed consent to participate in this study.

## Author Contributions

CD, MW-F, and PL-M designed the study. CD collected the data. TR conducted acoustic analyses. LF conducted perceptual analyses and mixed modeling. LF and PL-M drafted the manuscript. All authors edited and approved the manuscript.

## Conflict of Interest

The authors declare that the research was conducted in the absence of any commercial or financial relationships that could be construed as a potential conflict of interest.

## Footnotes

1 We ran an additional *t*-test comparing the MCC of the subset of familiar participants who did not speak Yorùbá to those unfamiliar and still found a large, significant effect of familiarity, *t*_(__58.74)_ = 3.18, *p* = 0.002, *d* = 1.16, ΔMCC = 0.211.
