# Supplementary material for: Perception of Nigerian Dùndún Talking Drum Performances as Speech-Like vs. Music-Like: The Role of Familiarity and Acoustic Cues
Source: Front Psychol. 2021 May 20;12:652673. doi: 10.3389/fpsyg.2021.652673 (PMC8173200; doi:10.3389/fpsyg.2021.652673)
Supplement: Supplementary file 1 [file Table_1.DOCX]

Supplementary Material

## Supplementary Figures


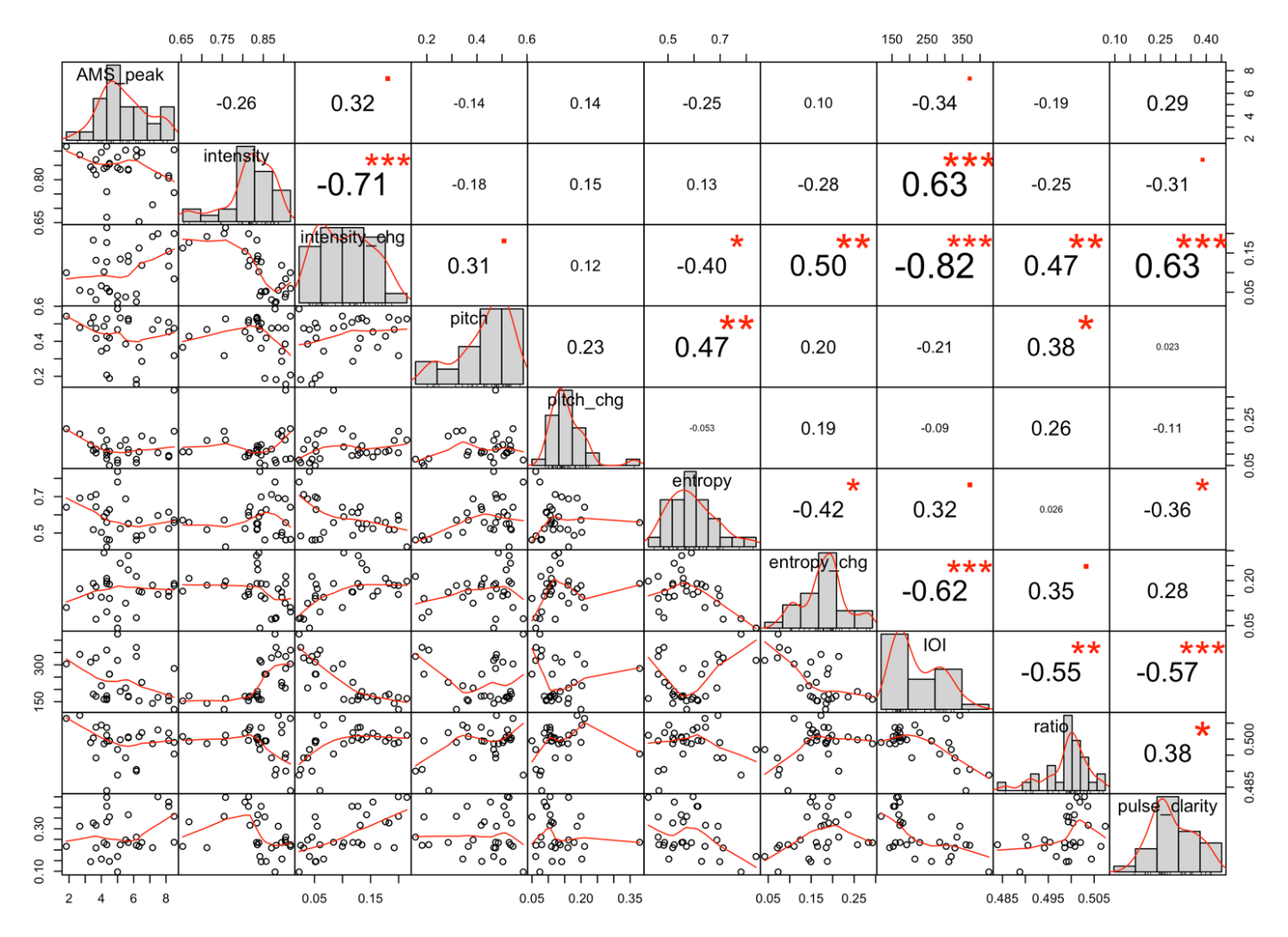


**Supplemental Figure 1.** Correlations between means of all acoustic features of interest. Histograms and the name of each feature are plotted along the diagonal. Pearson correlation coefficients and statistical significance are displayed in the upper right triangle (*** p < .001, ** p < .01, * p < .05, **^.^** p < .1). Bivariate scatterplots and a line of best fit are displayed in the lower left triangle.
